# From conformons to human brains: an informal overview of nonlinear dynamics and its applications in biomedicine

**DOI:** 10.1186/1753-4631-1-5

**Published:** 2007-07-05

**Authors:** Wlodzimierz Klonowski

**Affiliations:** 1 Institute of Biocybernetics and Biomedical Engineering Polish Academy of Sciences, Warsaw, Poland; 2 GBAF, Medical Research Center Polish Academy of Sciences, Warsaw, Poland

## Abstract

Methods of contemporary physics are increasingly important for biomedical research but, for a multitude of diverse reasons, most practitioners of biomedicine lack access to a comprehensive knowledge of these modern methodologies. This paper is an attempt to describe nonlinear dynamics and its methods in a way that could be read and understood by biomedical professionals who usually are not trained in advanced mathematics.    After an overview of basic concepts and vocabulary of nonlinear dynamics, deterministic chaos, and fractals, application of nonlinear methods of biosignal analysis is discussed.  In particular, five case studies are presented: 1. Monitoring the depth of anaesthesia and of sedation; 2. Bright Light Therapy and Seasonal Affective Disorder;  3. Analysis of posturographic signals;  4. Evoked  EEG  and photo-stimulation;  5. Influence of  electromagnetic  fields generated by cellular phones.

## 1. Background

An increasing importance of nonlinear dynamics for biomedicine is so evident that it even impressed the European Parliament. EP's report on physiological and environmental effects of non-ionising electromagnetic radiation bluntly states:

"It should be noted that difficulties sometimes experienced in attempts to independently replicate certain frequency-specific non-thermal effects are *actually to be expected*. For *in consequence of the highly non-linear, non-equilibrium nature of living systems*, even the slightest differences in the physiological state of the biosystems used and in the conditions obtaining in a particular experiment can, in consequence of deterministic chaos, assume singular importance. (...) Future EU-sponsored research should incorporate the following recommendations: (...) That systematic investigation be made (...) *whether any observed changes in power spectra are correlated with changes in the level of deterministic chaos*" (cf. [1], italics by the author).

Today's academic curricula still emphasizes mainly the basics of algebra and 'classical' linear XVIII–XIX century physics of 'static' systems that are in a state of equilibrium or close to equilibrium. On the other hand contemporary sciences as well as technology are heavily based on 'new' nonlinear physics that, in order to be understood requires a working knowledge of calculus and advanced mathematics beyond calculus. It is in fact nonlinear dynamics that applies to systems that are not in equilibrium – from a single protein macromolecule with intrinsic physical instability (in scientific literature called *Klonowski-Klonowska conformon *[2,3]) and self-oscillating cross-linked (bio)polymeric material [4,5], to nonlinear dose-effect relationships in medicine (called *hormesis*) [6], and human brain [7]. This article is an attempt to provide a comprehensive description of nonlinear dynamics in a way that does not require mathematical sophistication from the readers. I do hope that the exposition of nonlinear dynamics presented here would be useful to physicians, biologists, social scientists and other professionals who are involved in diverse fields of today's biomedicine but have not been specifically trained in advanced mathematics.

## 2. Fundamental concepts

### 2.1. Basic definitions

***System ***may be defined as an orderly working totality, a set of units combined by nature, by science, or by art to form a whole (cf. [8]). System is not just a set of ***elements ***but includes also ***interactions ***between both the system's elements and with the 'external world'. Interactions may be *static *(like rigid mechanical connections e.g. a set of reinforced concrete beams linked together to form skeleton system of a building) or *dynamic *i.e. through exchange of mass, energy, electric charge (like molecules of neurotransmitters or ion flows through channels in cell membrane) or through exchange of information (through e.g. electromagnetic fields like cellular phones and communication satellites in a GMS system or through acoustical waves like a group of individuals speaking one to another).

A living organism is an *open system*, 'pumped' with free energy from biochemical reactions, in a similar way to a TV-set being 'pumped' with free energy from electrical outlet. Electromagnetic fields (EMF) that interact with a TV-set carry extremely low energy; what EMF do carry is *information *that influence the system, so causing that either meaningful or just noisy images show up at the TV-screen. We call such effects *information interactions *(cf. [3]).

In physics *state *of a system in a given moment of time is characterized by values of *state variables *(at this moment). The minimum number of independent state variables that are necessary to characterize the system's state is called the number of *degrees of freedom *of the system. If a system has *n *degrees of freedom then any state of the system may be characterized by a point in an *n*-dimensional space with appropriately defined coordinates, called the *system's phase space*

***Process ***is defined as a series of gradual changes in a system that succeed one another (cf. [8]). Every process exhibits a *characteristic time*, *τ*, that defines the *time scale *for this process. As a consequence, for every process time should be measured in a form of non-dimensional quantity *t/τ*, expressed in units equal *τ *rather than in 'absolute' units like seconds or years. In the system's phase space a process is represented by a series of connected points called *trajectory*. *Attractor *is a subset of the system's phase space that attracts trajectories (i.e. the system tends towards the states that belong to some attractor). For example, in classical thermodynamics a closed system tends towards the equilibrium state while an open system tends towards a steady state.

***Signal ***is a detectable physical quantity or impulse (as a voltage, current, magnetic field strength) by which information can be transmitted from a given system to other systems, e.g. to a measuring device (cf. [8]). Before further analysis a signal is usually *sampled *with certain *sampling frequency*, *f*_*s*_. So, in computer memory each signal is registered in a form of *time series *while on computer screen or on paper it often looks like a continuous function. Signals generated by a system change with time in accordance with the processes occurring in the system, e.g. voltage measured on the scalp (EEG-signal) changes depending on processes in the brain. Signals generated by body organs or single cells are called ***biosignals ***e.g. brain produces EEG-signals, heart – ECG-signals, muscles – EMG-signals. Biosignals may be spontaneous or ***evoked ***by some external stimulus e.g. a flash of light.

***Noise ***is any unwanted signal that interferes with the desired signal (cf. [8]). Noise may be caused by undesired processes both in the examined system and in the measuring devices.

### 2.2. Nonlinear *vs*. linear

***Linearity ***in science means more or less the same as *proportionality *or *additivity*. In classical physics if one applies two times greater force the body will gain two times greater acceleration (Newton's second law); if one applies five times greater voltage difference to the ends of a resistor the current through that resistor will also be five times greater (Ohm's law). But it is well known that if the applied force or the applied voltage would be too high then the body or the resistor will be destroyed – linearity has its limits.

On the other hand linearity means that interactions between system's elements are negligible. If, for example, one mixes noble gases argon and neon, the properties of the resulting mixture will be a simple combination of properties of its components because interactions between atoms of noble gases are negligible. But if one mixes substances that interact strongly, for example can undergo chemical reactions with each other, the resulting new system consisting of reaction products have properties that cannot be easily determined solely from those of the initial components (substrates). For example, if one mixes hydrogen and oxygen in volume proportion 2:1 then a spark is enough and the gaseous mixture is transformed into liquid water of total volume about 1870 times smaller than that of the initial mixture. Such *emergence *of new (or emergent) properties is an obvious result of interactions between system elements. So, ***nonlinearity ***means more or less the same as *nonadditivity*.

*Reductionism*, a methodological attitude of explaining properties of a system through properties of its elements alone, may work only for linear systems. One cannot explain properties of water as a simple combination of properties of hydrogen and oxygen. Similarly, it is unreasonable to try to explain properties of the brain from properties of neural cells (neurons) alone. The same lack of plausibility of causal reduction appears to be true in social systems. For example, a group of several persons (even of 2 persons as in matrimony) has properties there are not a simple sum of properties of individuals. A crowd has some collective properties as well – quantity changes into a new quality.

Some systems have properties that depend more on the way *how *the elements are connected than on what the specific properties of individual elements are. For example, gels owe its 'gel-like' properties to the fact that practically all molecules in the system are connected into one single network rather than into a set of many big aggregates [9].

**Figure 1:**
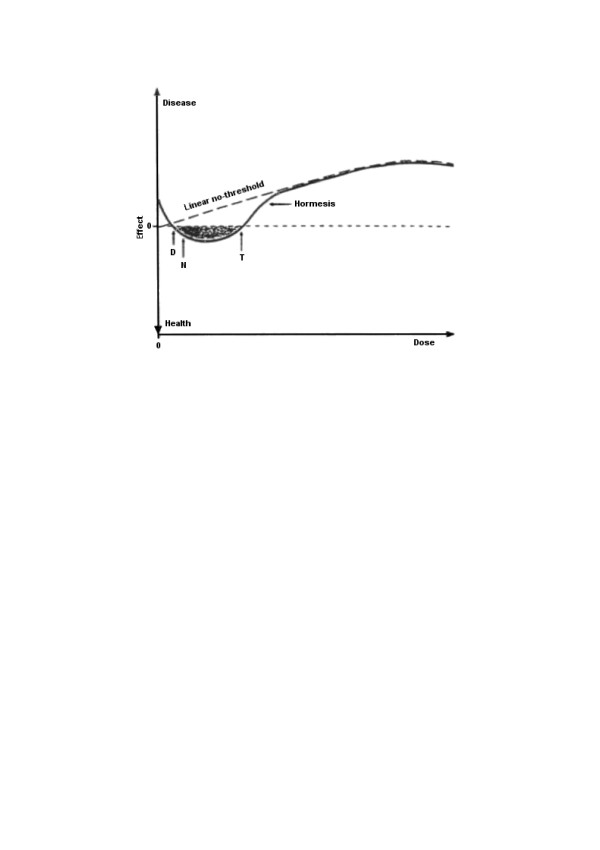
**Example of hormesis – biological response to chemical and physical agents**. Deficiency symptoms are caused by deficit of an agent (dose less than D); small doses (between D and T) are vital for good health (shaded area), doses higher than T cause toxic or other harmful effects. Dotted line represents linear no-threshold relationship, solid line represents hormetic dose-effect relationship (after [10])

In everyday life one often observes *hormesis*, a nonlinear dose-effect relationship (Fig. 1) without even noticing this phenomenon. It is well known that alcohol in large amount is a poison that can kill via inhibiting some processes, but in small amounts it acts as a stimulant. The same applies to effects of caffeine, nicotine, drugs. The problem is with the adjective 'small' – what for one individual is still a small stimulating amount for another may already be a lethal dose. There is no universal rule or a threshold between stimulating and poisoning doses. From our perspective though hormesis is a very common phenomenon that exemplifies the notion of nonlinearity, but unfortunately is often forgotten in medical data analysis,

In medical papers one can often find erroneous data interpretation that combines linearity with a *threshold*. However, a thresholdis incompatible with linearity as one can easily observe from an example of 'linear income tax' examined in the context of a 'tax-free amount' (Table 1).

**Table 1:** Example of 'linear' income tax with a threshold – everybody pays the same percentage (here 25 %) of the income exceeding tax-exempted amount ('threshold', here 10.000)

	**Without threshold**		**With threshold 10 000**	
**Income**	**Tax to be paid**	**% of income**	**Tax to be paid**	**% of income**

5 000	1 250	25	0	0
10 000	2 500	25	0	0
15 000	3 750	25	1 250	8.33
20 000	5 000	25	2 500	12.50
50 000	12 500	25	10 000	20.00

### 2.3. Far from equilibrium vs. equilibrium

Any process to occur needs a difference of some (generalized) potential. Thermodynamic ***equilibrium ***means a complete lack of differences between different parts of the system and, as a consequence, a complete lack of changes in the system – all processes are stopped.

'Living' states of any system are nonequilibrium states. In these states some properties (like concentrations, electrical charges, etc.) are unequally distributed, and those differences act as driving forces for all processes. Equilibrium, the unique state when all properties are equally distributed, is the state of 'death'. It is true not just for a single cell or an organism. The socio-economical system in which there are no inequalities between the people may not exist for a longer period of time – it decomposes like a dead organism. If those who had 'invented' communism knew thermodynamics they would understand that communistic system would necessarily fail, as we all have observed.

In the systems being close to equilibrium one can observe linear processes while in the systems being ***far from equilibrium ***processes are nonlinear. Life appears to be a nonlinear phenomenon that may not be explained solely by properties of components of living systems. Reductionism is fundamentally flawed because of *emergent properties *which appear in complex systems far from equilibrium.

Even in a single protein macromolecule, due to differences in time scales of processes involved in protein biosynthesis, one may observe intrinsically nonequilibrium structures called *Klonowski-Klonowska conformons *(in proteins with short turnover time, cf. [2,3]). Another example are spatio-temporally organized networks of physicochemical processes that may be identified with *intracellular dissipative structures *[2], defined generally as the dynamics organization of matter in space and time kept far from equilibrium by a continuous dissipation of free energy; nonequilibrium structures, like sub-cellular sol-gel dissipative network structures may be built up even of subunits that taken separately are in thermodynamic equilibrium (cf. [4,5]).

### 2.4. Nonstationary *vs*. stationary

***Stationarity ***of a signal means that the signal, and so the time series representing this signal, has the same mean and variance throughout. Stationarity does not mean constancy – stationary signal may be changeable like e.g. voltage in alternating current outlets.

***Nonstationarity ***means that signal's statistical characteristics change with time. In statistics *nonstationary mean time series *refer to time series whose average or mean value is not constant, like in time series with trends or seasonalities; *nonstationary covariance time series *are time series whose correlation or covariance changes with time (cf. [11]).

Biosignals are usually nonstationary. For example, EEG-signal recorded from a scalp electrode is influenced by different deeper brain structures, each 'transmitting' with different and changeable intensity; so, in a fraction of a second the main source of the registered signal often moves from one brain structures to another. And if source of a signal changes with time then the signal is obviously nonstationary.

Example of stationary and nonstationary signals and their analysis is given below in 4.3.

### 2.5. Stochastic *vs*. deterministic

***Deterministic ***means more or less the same as *predictable*. If a system is deterministic one can predict the system's future states. Deterministic systems are either characterized by sufficiently small number of degrees of freedom or some state variables are of negligible importance compared to those of the greatest importance. For example, in a system consisting of a mixture of different isotopes there may be 'quick' isotopes for which characteristic times of their decay, *τ *(these isotopes' half-life, *τ*_1/2_) is extremely short comparing with the time of observation *Δt*, 'slow' isotopes with *τ*_1/2 _of the same order of magnitude as *Δt*, and 'crawling' isotopes with *τ*_1/2 _much greater than *Δt*; so, in a period of time *Δt *concentration of 'crawling' isotopes remains practically constant, while concentration of 'quick' isotopes reach very quickly its steady state value (steady state value is also constant, but it is a dynamic constancy – the 'inflow' and 'outflow' are equal while both being non-zero). If one is interested in modelling this system to predict its state after time of the order of *Δt *the system will behave as one with the number of degrees of freedom reduced to the concentrations of 'slow' isotopes. Deterministic systems are modelled by linear ordinary differential equations (ODE, cf. below). But to use a model of a deterministic system one needs to know **exactly **its initial conditions, i.e. the exact values of state variables at the initial moment *t *= *t*_0_, and **exact **values of systems parameters.

***Stochastic ***means *nondeterministic*, *nonpredictable*. Stochastic system has a very big number of degrees of freedom of similar importance. So, the difference between deterministic and stochastic system is rather quantitative (number of equally important degrees of freedom) than qualitative. Stochastic systems are modelled using probability theory. Nonpredictivity may also result from practical impossibility of even indicating all parameters that influence the process, not to speak about giving exact values of all parameters and initial conditions, as e.g. in tossing a coin. In colloquial speech, stochastic systems used also to be called *chaotic*.

## 3. Nonlinear dynamics, deterministic chaos, fractals

### 3.1. Sensitivity to initial conditions

***Nonlinear dynamics ***is the theory of nonlinear systems and processes, those where result is not proportional to the cause. Everybody knows situations when even a very small difference in applied force causes completely different results. Nonlinear dynamics helps us to study and to generalize such cases. However, it often needs much more advanced mathematics than in the case of classical linear dynamics.

Nonlinear dynamics includes theory of ***deterministic chaos***. *Chaotic systems *behave like there were stochastic but in fact they are deterministic. They show predictability in a short-time-scale but non-predictability in a long-time scale due to extremely *high sensitivity to initial conditions and to system's parameters *(cf. below).

Imagine a train on a track that leads to a railway station. Before the station there is an old fashion mechanical points (switch) and the single track splits into two, so that at the platform there are two parallel tracks. Crossing attendant moves the switch lever and depending on the position of this lever heavy train pulls on one track or on the other; when the train leaves the station two tracks run parallel for some distance but then they diverge without any further connection and one leads to London while the other leads to Rome. A small change (moving of a switch lever) at certain moment causes very large difference in trajectories of the train in the subsequent moments.

### 3.2. We integrate differential equations without even knowing it

Surprisingly, we all integrate differential equations. We often are like a Monsieur Jourdain from Moliere's "Le Bourgeois Gentilhomme" who said (II.iv): "Good heaven! For more than forty years I have been speaking prose without knowing it."

*Ordinary differential equation (ODE) *of the first order that circumscribes dependence of the velocity of changing of state variable *x *with time *t*

*dx/dt *= *v(t)*

where *dx/dt *is the *derivative *of state variable *x *over time *t *and *v(t) *is a function of *t*, needs to be *integrated *to obtain *x(t)*, i.e. the dependence on time of the state variable itself. In a special case when the velocity *v *is constant equation (1) can easily be integrated

*x(t) *= *v*·*t *+ *x(0)*

where *x(0) *is the *initial condition *– the value of the state variable *x *at the moment *t *= *0*. But in such simple cases one does not need to know theory of differential equations. Everybody can easily calculate that if at 7:30 a car starts from the place already 100 km from the beginning of the journey and moves with constant average velocity of 90 km/h then at 9:00 it will be 235 km from the starting point.

### 3.3. Example of deterministic chaos

For systems with several degrees of freedom one may write ODE like (1) for each state variable. Usually right hand sides of such equations do not depend directly on time but are functions of the state variables; if right hand sides are nonlinear functions, i.e. include products of different state variables and/or single state variables squared or in higher powers multiplied by some numbers (parameters), they are called *quasilinear ordinary differential equations (QLODE)*. Dynamical system may be modelled by system (in mathematical sense) of QLODEs, i.e. a set of equations that have to be solved all together.

If to model a system one needs three or more QLODEs this system may show deterministic chaos i.e. extremely high sensitivity to initial conditions and to system's parameters. As an example let us consider so called Lorenz equations (cf. [12]):

*dx/dt *= -*ax *+ *ay*

*dy/dt *= *bx *- *y *- *xz*

*dz/dt *= -*cz *+ *xy*

with two sets of parameters:

*a *= 10; *b *= 28; *c *= (8/3)

*a *= 10; *b *= 28; *c *= (8008/3000)

and three sets of initial conditions:

*x(0) *= 0.999; *y(0) *= 0.999; *z(0) *= 0.999

*x(0) *= 1.000; *y(0) *= 1.000; *z(0) *= 1.000

*x(0) *= 1.001; *y(0) *= 1.001; *z(0) *= 1.001

The first surprise is that the results show very irregular behavior while inspecting equations (3) no one could directly predict such a strange behavior of the solution – because of the simplicity of the equations one would rather expect smooth, regular behavior. So, anybody using such models (in chemistry, biology, engineering, economics, business, and humanities) should be aware of possible pitfalls hidden in even simple QLODEs.

**Figure 2:**
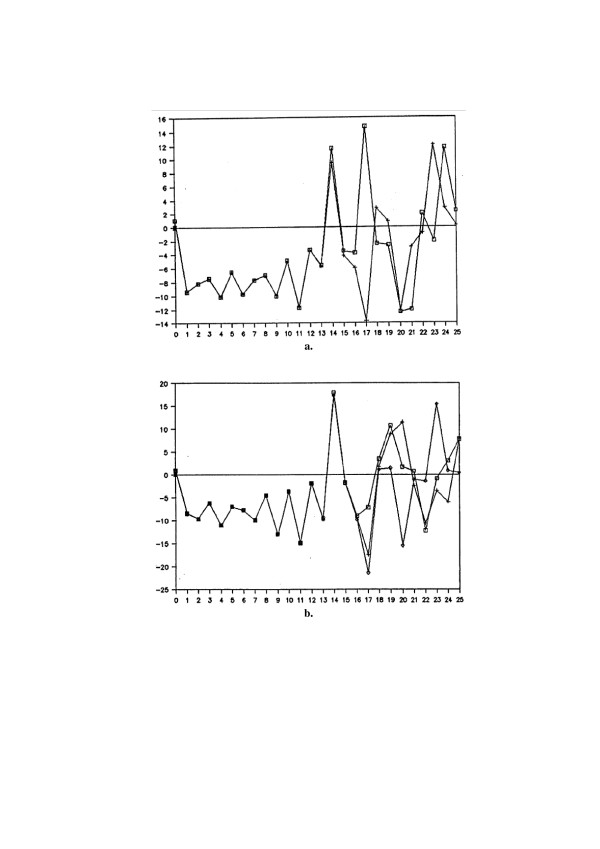
**Example of deterministic chaos – sensitivity of solution of Lorenz equations **(3) illustrated by dependence of *x *on *t*; state variables *y *and *z *show similar chaotic behavior. **a. **sensitivity to system parameters for initial conditions (5a), solutions for parameters (4a) (crosses) and (4b) (squares), respectively; **b. **sensitivity to initial conditions for system parameters (4a), solutions for initial conditions (5a) (crosses), (5b) (squares), (5c) (diamonds) respectively.

Fig. 2a shows one 'face' of behavior of chaotic system – its **extreme sensitivity to small changes in system parameters**. When parameters of Lorenz equations (3) are very slightly changed (from (4a) to (4b), that is only one of three parameters, *c*, is increased one tenth of a per cent, while the initial conditions remain unchanged, equal (5b)) one observes that at the beginning two solutions remain practically identical; then, quite suddenly an unpredictable very big difference in solutions does appear.

Another 'face' of the behavior of chaotic system – its **extreme sensitivity to small changes in initial conditions **– is shown on Fig. 2b. When the system parameters (4) remain unchanged (equal (4a)) but initial conditions are changed from (5b) to (5a) or (5c), i.e. one tenth of a per cent, again at the beginning three solutions remain practically identical, and then quite suddenly an unpredictable very big differences in solutions does appear.

So, even a very small change of initial conditions and/or of system parameters may bring dramatic changes in a long-time behavior of the solution of equations (3). The obtained results are not some artifacts introduced by numerical integration, but are really the inherent properties of the considered QLODEs. If such simple set of equations shows such a strange behavior of solutions one can imagine what is possible with models often containing dozens of equations with dozens of parameters. And one should remember that in a computer real numbers like (8/3) are always represented with some approximation, often out of control of the user.

Chaotic systems are inherently connected with ***fractals ***and ***fractal geometry***. When represented in a phase space chaotic systems shows so called ***strange attractors ***– attracting subsets of dimension expressed by a non-integer number (cf. [13]).

Because living systems are far from equilibrium inherently nonlinear systems nonlinear models of living systems and nonlinear methods of biosignals' analysis are much more appropriate than 'classical' linear methods.

## 4. Nonlinear methods of biosignal analysis

### 4.1. Shortcomings of linear methods of biosignal analysis

Spectral methods such as Fast Fourier Transform (FFT) may give very misleading results. E.g. if in a measured signal one observes regular waves of frequency 12 Hz with amplitude modulated with frequency 1 Hz then Fourier decomposition of this signal leads to two components, each of amplitude equal half of that of the analyzed signal, with frequencies 11 Hz and 13 Hz respectively:

*[2cos(2π*·*t)]sin(2π*·*12t) *= *sin(2π*·*11t) *+ *sin(2π*·*13t)*

So, the basic frequency of the analyzed signal (12 Hz) does not appear in the Fourier spectrum.

In *Fourier *analysis (FFT) *vocabulary *(i.e. the set of basic functions analyzed signals are decomposed onto linear combinations of) is limited to the set of sines and cosines of different frequencies.

In *Wavelet Transform *(WT) vocabulary may be much bigger, consisting e.g. of Gabor's functions, i.e. sines modulated by Gauss functions, depending not only on frequencies but also characterized by different time scales and shifted in time.

In *Matching Pursuit *(MP) method vocabulary includes generalized functions like Dirac's *δ*-function.

So, WT and MP have better accuracy but at the same time show much bigger ambiguity in signal decomposition.

Methods borrowed from nonlinear dynamics and deterministic chaos theory, in particular fractal and symbolic methods of analysis in time domain, are well suited for real-time biomedical applications.

### 4.2. Linear methods are rooted in medical tradition, nonlinear methods are not

Living systems are complex, nonlinear, and operate far from equilibrium. That is why nonlinear methods of contemporary physics are much more appropriate to model and analyse living systems and biosignals generated by them than linear methods traditionally used in medicine, like FFT or WT. But spectral methods are still widely used just because of tradition. PCs with FFT implemented already in EEG-data acquisition software were quickly accepted in clinical environment because FFT gives physicians exactly what they have been using – spectrum of waves with frequencies up to 35–45 Hz. But no generators of waves in different 'bands' observed in EEG have ever been localized in the brain, so spectral characteristics are just a way of description of EEG-signals.

While it is obvious that the higher is the frequency of a wave the more information it may carry and neuroscientists did find that some tasks, like e.g. face perception, elicit frequencies up to 250 Hz when the stimuli are processed by human brains [14] frequencies exceeding roughly 70–90 Hz are filtered out by EEG-data acquisition systems hardware, and so are very low frequencies smaller than 0.5 Hz. From such filtered signals, according to Nyquist Theorem, one cannot recover Fourier components with frequencies higher than 35–40 Hz. When EEG was registered on a moving paper tape (1.5, 3.0, or 6.0 cm/sec depending on the system) doctors could count number of pen sways (wave ridges) between two vertical lines 3 cm away; however, when that number exceeded 25–30 it was nearly impossible to distinguish the signal from noise because of the very thickness of a line drawn by the pen; on the other hand, frequencies below 3.0 Hz were also practically imperceptible by a naked eye, while waves with frequencies 8–12 Hz ('alpha waves') were most easily noticed. And this tradition is still alive despite the fact that EEG-signals are now registered numerically by computers.

The time series obtained from biological systems such as human brain are invariably nonstationary because of different time scales involved in the dynamical process; dynamical parameters are sensitive to the time scales and hence in the study of brain one must identify all relevant time scales involved in the process to get an insight in the working of brain [15]. Biosignals generated by living systems are ***'3N'***– ***Non-stationary***, ***Non-linear***, ***Noisy***. Nonlinear methods that assess signal complexity, like Higuchi's fractal dimension method, may be used for EEG (and other biosignals) analysis no matter if the signal itself is chaotic, deterministic, or stochastic, also when it is nonstationary and noisy. But still nonlinear methods are used only in research and not in everyday clinical practice because these methods are not yet rooted in medical tradition. While linear methods, as we have shown above, have serious drawbacks, nonlinear methods may be used to analyse changes in EEG-activity due to anaesthesia or to the applied chemo-, photo- or magneto-therapy, or due to changes of physiological state, e.g. because of falling asleep and passing through subsequent sleep stages.

### 4.3. Higuchi's fractal dimension in time domain

As an example of nonlinear methods we take Higuchi's fractal dimension in time domain [16]. It is, in fact, fractal dimension of the curve representing amplitude of the signal under consideration on a plane as a function of time. So, it is always between 1 and 2, since as everybody knows a simple curve has dimension equal 1 and a plane has dimension equal 2. Imagine a classical registration device that records a signal like EEG by drawing with a pen a curve on a moving paper tape. Such a curve may in some degree fill out the plane (here the tape surface); if the device registers pure noise the plane will be filled out (smeared) completely. Higuchi's fractal dimension, *D*_*f*_, or rather its fractional part over 1, measures the 'degree of filling out' the plane by the curve, that is at the same time a measure of ***complexity ***of the signal represented by this curve. Higuchi's fractal dimension should not be confused with fractal dimension of an attractor in the phase space.

**Figure 3:**
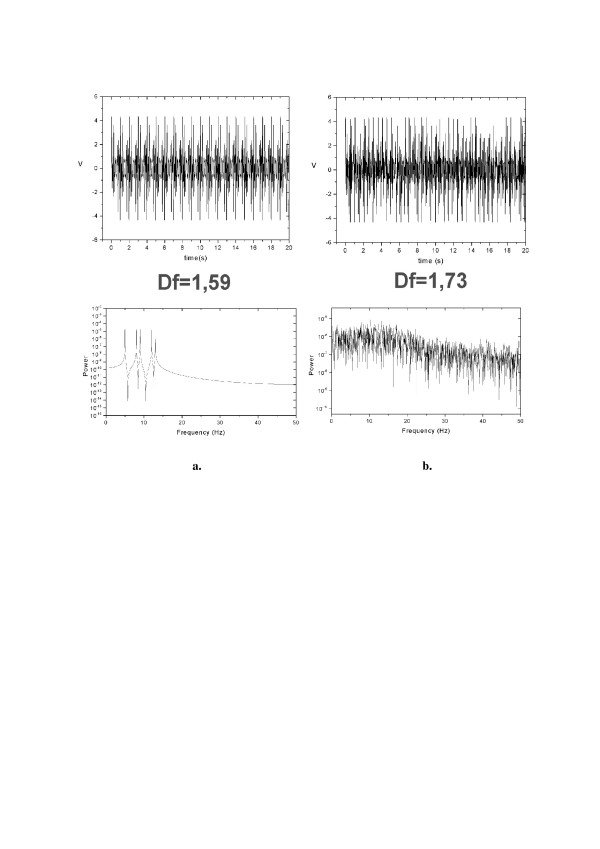
**FFT applied to similar stationary (upper a.) and nonstationary (upper b.) signals **gives dramatically different results (bottom), while application of Higuchi's algorithm gives quite similar values of average fractal dimension, *D*_*f*_, of both signals.

*D*_*f   *_may be calculated in a time window containing so few as ca. 100 data points and the window can be moved along the signal, so giving substantial compression of data. Using moving window one obtains *running fractal dimension D*_*f*_*(t) *that shows changes of the signal's complexity in time. Higher values of *D*_*f *_correspond to presence of higher frequencies in the signal's Fourier spectrum (cf. [17]). While running fractal dimension may be used to characterize short-lasting phenomena like eye blinking, the mean fractal dimension value averaged over longer period of time also serves as a meaningful characteristic of studied biosignals. Moreover, while linear methods, like FFT or WT, work properly only for stationary signals, Higuchi's methods may be used to characterize nonstationary signals too (Fig. 3) – signals shown at the upper part look slightly different and their mean fractal dimensions are also only slightly different but their power spectra (the lower part) are immensely different; it is so because the second signal is nonstationary.

Signal presented in Fig. 3a upper part was 'synthesized' by adding together 5 simple sinusoidal signals of different frequencies; when this artificial signal is analyzed using FFT we can clearly see in its spectrum those 5 components (Fig. 3a bottom). Signal presented in Fig. 3b upper part was obtained from this shown on Fig. 3a by repeating several times the following procedure: 1. choosing randomly a short signal epoch; 2. removing the chosen epoch; 3. 'stitching together' two remaining parts of the signal. In the place of such a stitching 'jump' of signal's amplitude usually arises. This procedure is in fact very similar to artifacting of EEG-signal. One can observe that after such a procedure it is not possible to see in the signal's spectrum the 5 components of which the initial stationary signal was composed (Fig. 3b bottom). So, despite the fact that a raw EEG-signal is nonstationary, artifacting usually introduces additional nonstationarity and FFT-analysis of 'artifact-free' EEG may lead to very erroneous results. In most cases application of nonlinear methods like Higuchi's fractal dimension analysis are much more appropriate.

It is important to stress that Higuchi's fractal dimension does not show what is the 'real character' of the system that generated given biosignal – is it deterministic, chaotic, or stochastic. The method may be applied to any signal and should be treated as a tool to demonstrate *relative changes *in the signals, in particular in the same signal of the given person *'before' *and *'after' *(for example EEG of the patient before and after administration of a drug, before and after applying photo-stimulation etc.) or the same signal in different physiological states (e.g. in wakefulness and different sleep stages).

Transforming original biosignal into running Higuchi's fractal dimension *D*_*f*_*(t) *allows to reduce the amount of data without losing diagnostically important information – changes in *D*_*f*_*(t) *reflect changes in brain activity. Because *D*_*f*_*(t) *compresses long epochs of raw data into much smaller *D*_*f*_-epochs, such that the eyes can take in the whole picture at once instead of viewing the original record 'page after page', it may make easier for doctors to choose which fragments of record are important and should eventually be further checked using other methods.

**Figure 4:**
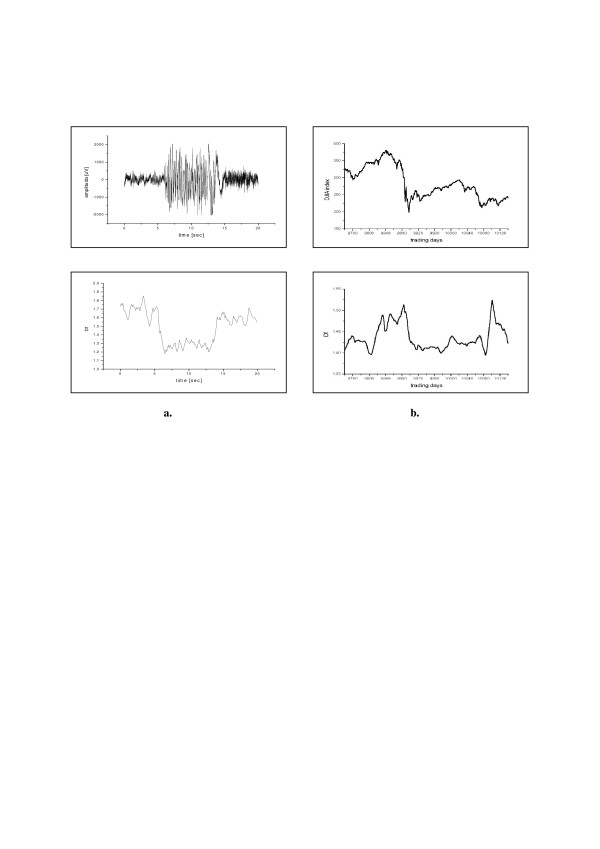
**'Epileptic seizures' in 'economic organism'**. **a. **EEG-epoch showing an epileptic seizure (top) and its fractal dimension (bottom); **b. **Dow Jones index from the period of 'big crash' (top) and its fractal dimension (bottom).

Higuchi's method may also reveal quite unexpected similarities in very different systems. For example, analysis of a 'signal' generated by an 'economic organism' – time series of Dow Jones index during the period of 'big crash' – shows very similar image of changes in Higuchi's fractal dimension as EEG-signal during epileptic seizure (Fig. 4, cf. [18]).

## 5. Nonlinear Biomedical Physics – examples of applications

### 5.1. Monitoring the depth of anaesthesia and of sedation

Brain electrical activity in patients was measured continuously with an A-2000 BIS Monitor (software version: XP, Aspect Medical Systems, Newton, MA, USA) and bispectral index (BIS) was recorded every 10 seconds. The bispectral index is commonly accepted as a measure of hypnosis during anaesthesia, but the algorithms the BIS Monitor uses are not in public domain. In addition, depth of anaesthesia was continuously tested and classified by a specialist-anaesthesiologist to six OAA/S (Observer's Assessment of Alertness and Sedation) levels; patients were judged to be conscious if the OAA/S score was between 3 – 5 and unconscious if the OAA/S score was less then 3 (cf. [19]).

**Figure 5:**
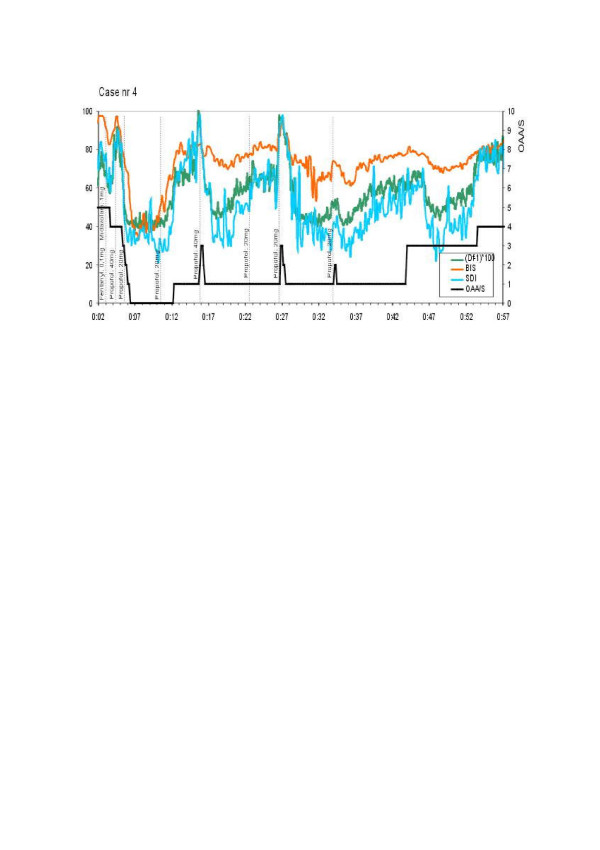
**Measuring the depth of sedation (cf. [19]) – fractal and symbolic dynamics methods of EEG analysis give similar results as BIS method**. In this case sedation has been employed to secure adequate comfort for 65-year-old male patient during long, unpleasant procedure (colonoscopy with polypectomy). Sedation has been controlled according to the BIS (target BIS value between 60 and 80) with intermittent boluses of propofol. At 16. and 27. minutes of the study sedation was lightened to level 3 in OAA/S score. Record showed that at those moments (*D*_*f *_- 1)*100 rose rapidly to rich the highest value. BIS increased only once (at 27. minute) while at 16. minute remained unchanged. Awakening can be predicted by rise of the (*D*_*f *_- 1)*100 towards its highest value. Exact dosage and timing of drugs administration are also shown.

We analyzed EEG-signals post-operatively. The results were averaged every 10 s for epochs 30 s long. Since 1 ≤ *D*_*f *_≤ 2 the fractal dimension value has been presented as (*D*_*f *_ - 1) ^.^ 100 to adjust the scale for better comparison with BIS (Fig. 5). We demonstrated that the fractal dimension corresponds to the depth of anaesthesia and we applied for a patent for this new method of anaesthesia monitoring. In addition we have used a new symbolic dynamics method to calculate another measure of the depth of anaesthesia, called *SDI *([21], cf. also [22]), but because this methods needs more complicated explanations we will not present this method here.

### 5.2. Bright Light Therapy and Seasonal Affective Disorder

Therapy of some pathological states may be based on techniques controlling chaos in the brain. Chaos in the brain may be controlled using different chemicals or using physical fields. It is phototherapy, that is the therapy using visible light, which is applied in patients with Seasonal Affective Disorder (SAD). As day light began noticeably decreasing in the fall many people may develop SAD. They feel very sedentary, and often sluggish. Physical activity diminishes and hypersomnia can develop. People suffering from SAD experience these and other symptoms to such a degree that they feel unable to function normally. About five times as many people may suffer from "winter doldrums," a sub-clinical level of SAD, than from a level of clinical severity. Toward spring with lengthening daylight hours, the number of affected people began to decline.

The most established treatment for SAD is so called Bright Light Therapy (BLT) which involves exposure to intense levels of white light (10,000 lux illumination) under controlled conditions. Its therapeutic mechanism is still unclear. We have searched for such quantitative characteristics of EEG in patients with SAD which may be relatively easily calculated, may help doctors in assessing therapy impact on the patient, and eventually help in diagnostics.

**Figure 6:**
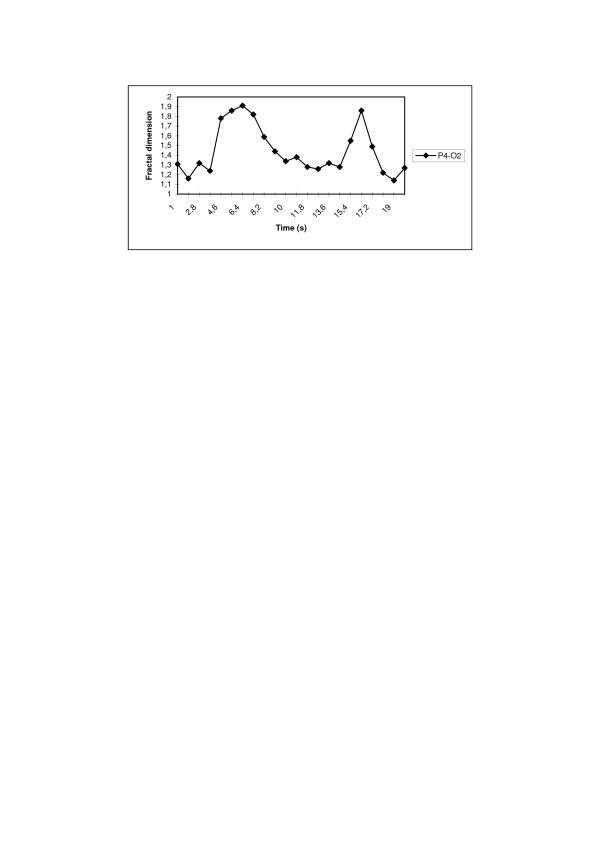
**Fractal dimension of 19 seconds EEG-epoch (P4-O2) with eyes-opening (in 4. s.) and eyes-closing (in 15. s.)**.

We analyzed EEG from 10 SAD patients. The data were collected before and after BLT. The second recording was provided 2 weeks after the treatment. For every patients we analyzed epochs of duration approx. 20 second starting about 5 seconds before eyes-opening and ending about 5 seconds after eyes-closing. For medical assessment of the patients the psychological Hamilton Depression Rating Scale (HDRS) was used. We have demonstrated using fractal dimension method that in patients suffering of SAD the 'relaxation' of *D*_*f *_after eyes-opening/eyes-closing is much slower than in healthy subjects [23]. When an eyes-opening event occurs fractal dimension of EEG-signal grows from 1.1–1.3 to 1.5–1.6 in the occipital channels and even to 1.8 in the frontal channels – this increase is denoted Δ_o_; when eyes remain open fractal dimension diminishes, to rise again when an eyes-closing event occurs; when eyes remain closed, it diminishes again – this decrease is denoted Δ_c _(Fig. 6 and Table 2). We define Δ_o_/Δ_c _as *open-/closed-eyes fractal dimension ratio (FD-ratio)*. We observed that in EEG of healthy subjects this ratio is close to 1. For SAD patients the *FD-ratio *was compared with HDRS before and after BLT (Table 2).

**Table 2:** *FD*-ratio and HDRS for SAD patients before and after Bright Light Therapy

**Patient**	**Δ_o_**	**Δ_c_**	**Δ_o_/Δ_c_**	**HDRS (before)**	**Δ_o_**	**Δ_c_**	**Δ_o_/Δ_c_**	**HDRS (after)**
1	0.78	0.82	0.9	high	0.75	0.80	0.94	low
2	0.26	0.41	0.6	high	0.47	0.47	1.0	low
3	0.17	0.42	0.4	high	0.37	0.25	1.5	low
4	0.40	0.56	0.7	high	0.17	0.17	1.0	low
5	0.68	0.75	0.9	high	0.20	0.20	1.0	low
6	0.73	0.55	1.3	high	0.54	0.51	1.1	low
7	0.53	0.64	0.8	high	0.42	0.41	1.0	low
8	0.37	0.48	0.8	high	0.26	0.25	1.0	low
9	0.39	0.56	0.7	high	0.46	0.41	1.1	low
10	0.22	0.59	0.4	high	0.36	0.36	1.1	low

For patients with high HDRS *FD*-ratio differs from 1.0; for patients for whom HDRS diminished after phototherapy *FD*-ratio 'normalizes' – it becomes closer to1.0. The differences between the columns 4 and 8 are statistically significant. So, *FD*-ratio may be used for assessment of BLT results in patients with SAD since it highly correlates with patients' assessment based on HDRS (Table 2). The method is easy to implement because it needs only short one channel EEG epochs and it is also quick – analyzing of a 20 seconds epoch requires only few seconds.

**Figure 7:**
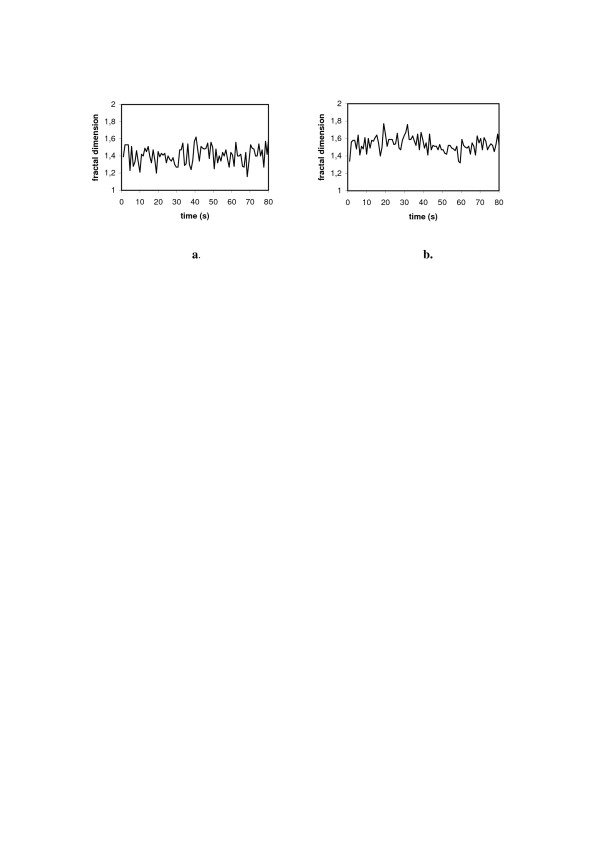
**Fractal dimension of EEG-signal for 80 sec. epochs (channel P3-O1) for a patient with SAD**. **a. **before BLT; **b. **after BLT.

We have also demonstrated that in patients suffering of SAD mean *D*_*f  *_of EEG-signal is smaller than in healthy subjects. BLT makes the mean value of *D*_*f   *_in those suffering with SAD to increase (Fig. 7, cf. [24]).

### 5.3. Analysis of posturographic signals

**Figure 8:**
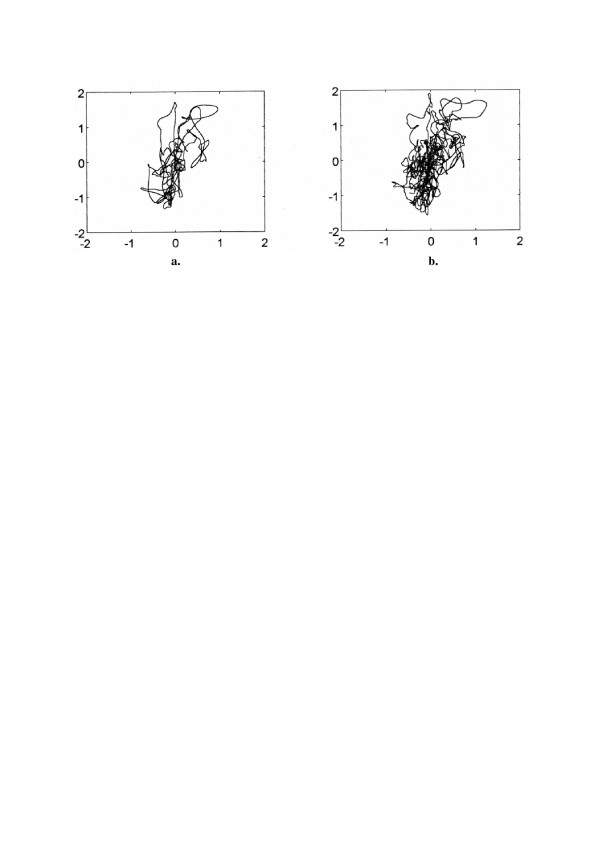
**Example of COM (a.) and COP (b.) signals recorded simultaneously **in a subject 74 years old during quiet stance in a 2-minutes trial; displacements measured in millimeters.

Stability of the upright posture is defined by a position of the center-of-mass (COM) in relation to the base of support. The position is approximated in static condition by the center of foot pressure (COP). Experimental data in posturography typically consist of *x *(anteroposterior, AP) and *y *(mediolateral, ML) components of COP and COM measurements. Analysis of complex oscillations exhibited by COM and COP may provide better understanding of postural control. In particular, the comparison of COP and COM displacements during quiet stance in elderly subjects while standing with eyes open and eyes closed can be used to evaluate differences in postural stability (Fig. 8).

**Figure 9:**
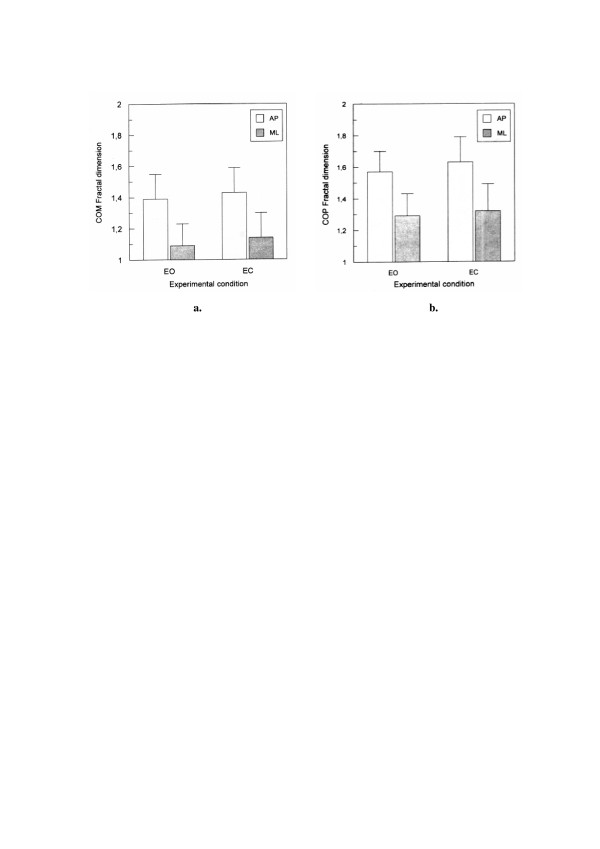
**Fractal dimensions (mean values with standard deviation bars) of COM (a.) and COP (b.) signals **in the elderly subjects during quiet stance with eyes open (EO) and eyes closed (EC); AP – for anteroposterior displacement; ML – for mediolateral displacement.

The body sway of 12 healthy elderly subjects (mean age 71.5 ± 3.6 years) was recorded during quiet stance; subjects were instructed to stand barefoot in a comfortable stance. The task was performed in two experimental conditions : with eyes open and eyes closed [25]. The postural sway was assessed by measuring COP and COM signals that were registered in the form of time series. Fractal analysis of data was applied for the evaluation of postural control system in these subjects (Fig. 9).

Higuchi's fractal dimensions of COM displacement signals were significantly smaller than these of COP. Eyes closure results in an increase of the postural sway, accompanied by an increase of fractal dimension – elimination of the visual feedback univocally causes increased chaos in COP. Higher fractal dimension in the AP direction indicates higher tendency for instability in this direction.

In our studies validity of the method was confirmed by comparing two major posturographic signals: COP and COM. It is well documented that postural stability declines with age; this decline seems to be accompanied by an increase of chaos in the postural signals. We postulate that COP signal in the aged is more chaotic.

Higuchi's fractal dimension method allows reliable evaluation of postural stability changes. It is useful and sensitive in evaluation of age-related decline of the postural stability and may also be useful for evaluation of pathological postural stability changes.

### 5.4. Evoked EEG and photo-stimulation

**Figure 10:**
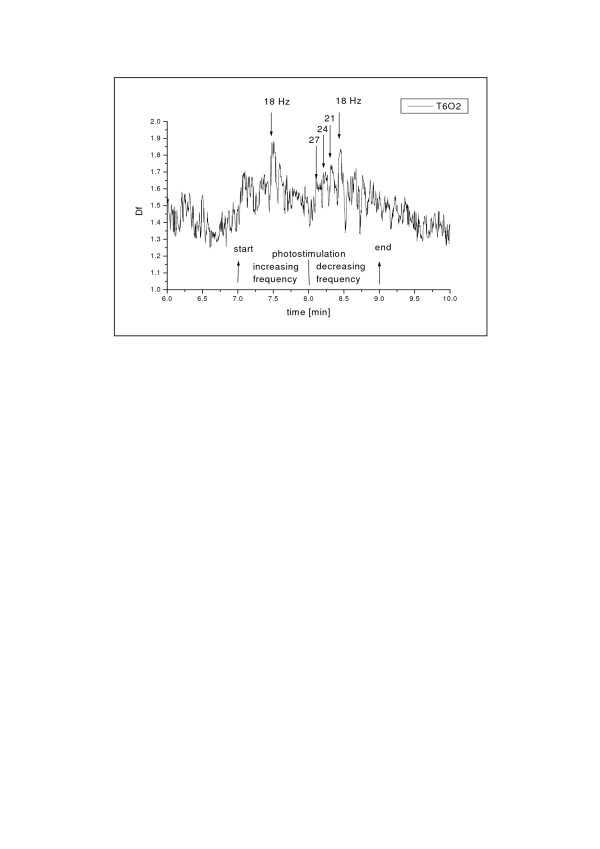
Fractal dimension at T6-O2 channel during photo-stimulation of a healthy subject.

Higuchi's fractal dimension may also be used for analysis of biosignals evoked by external stimuli, e.g. of EEG evoked by photo-stimulation. Photo-stimulation is performed in routine EEG-examinations. It consists of two sequences, the first in increasing frequency order (from 3 Hz to 27 Hz every 3 Hz), and the second in decreasing frequency order. Each stimulus consists of light flashing for 5 seconds with given frequency; there is one-second break between subsequent stimuli. Fig. 10. shows fractal dimension of evoked EEG-signal recorded on T6-O2 channel in a healthy subject [26]. One can notice clear dependence of fractal dimension on frequency of photo-stimulation with the maximum for 18 Hz. Higher frequencies cause more rapid changes in fractal dimension value than lower frequencies. In power spectra of the evoked EEG one cannot notice practically any relative differences for various frequencies of stimulation that are so clearly noticeable in fractal dimension [26]. Therefore, the fractal dimension is more revealing measure of the phenomenon.

### 5.5. Influence of electromagnetic fields generated by cellular phones

Assessing information effects of electromagnetic fields on living organism is much more complicated than that of energetic (thermal) effects. Exposition to EMF of identical parameters may cause very different reactions in different persons; even reactions of the same person may differ depending on the person's psycho-physiological state.

With wider spreading of cellular phones it becomes more and more obvious that EMF generated by these phones may have rather adverse effect on users' health [26]. Now companies offer devices that are said to level down such adverse effects, so called *neutralising protective devices*, NPD. While testing such an NPD and using classical spectral methods of analysis Spanish scientists found in all tested persons statistically significant differences in spectral power of slow EEG-waves (delta and theta) as compared with the power of the same waves in basal EEG (i.e. when the cellular phone was 'off'), while when the phone used was equipped with the NPD these differences were much lower [27].

**Figure 11:**
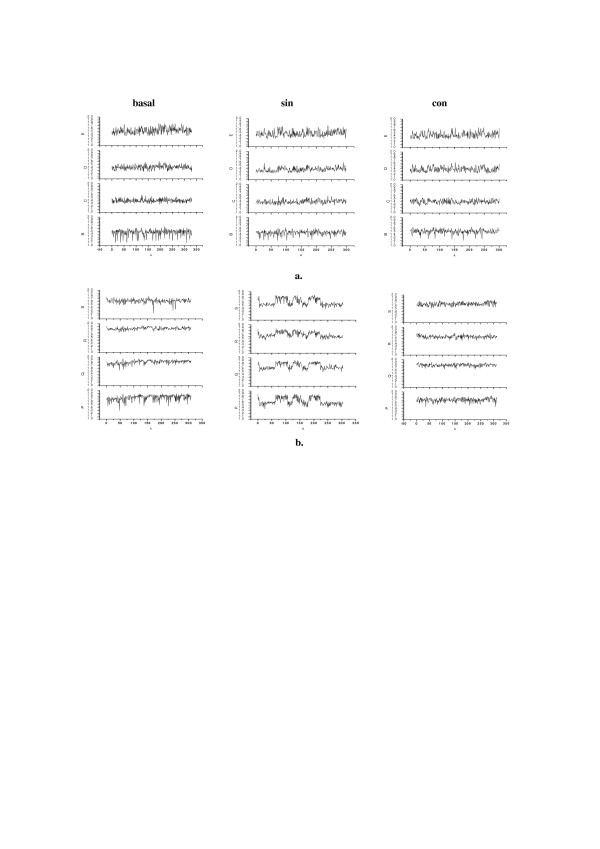
**Higuchi's fractal dimension of 300 sec. long epochs of EEG-signals**. **a. **for a person who does not show sensitivity to EMF;** b.** for a person (hyper)sensitive to EMF; **basal **– phone at place but not in use; **sin **– in use, without NPD; **con **– in use, with NPD; registered on 4 channels (respectively, in each column upside down): T6-O2; T4-T6; F8-T4; Fp2-F8 (from back to front of the head).

Analysing the same data while using methods of nonlinear dynamics, in particular Higuchi's fractal dimension method, only in 1 out of 8 tested persons we found clear differences in EEG-signals recorded when the person used cellular phone without the screening device, NPD (*sin*), as compared with EEG recordings for the same person when the cellular was 'off' (*basal*), or while using the phone equipped with the tested NPD (*con*). Only for this person running Higuchi's fractal dimension, *D*_*f*_, of EEG signals shows changes characteristic to sensitivity to electromagnetic fields (cf. [30]) (Fig. 11).

About 15% of population may belong to a *high-risk *(*hyper-sensitive*) group of users of cellular phones (at least of certain models). Proposed method may serve for quick and easy assessment of *individual susceptibility *to EMF used in mobile communication as well as for testing of different cellular phones models for their *certification *by the appropriate institutions.

## Conclusion

Until recently quantitative computerized EEG-signal analysis was based primarily on linear theory. But developments in nonlinear dynamics and deterministic chaos theory have considerably altered our perception and analysis of many complex systems, including the brain. Additional information extracted from EEG by methods of nonlinear analysis may increase the sensitivity of electrophysiological methods. Quantitative descriptors of EEG-signal adapted from nonlinear dynamics (information dimension, correlation dimension, Lyapunov exponents etc.) enable better assessment of various spontaneous or evoked, normal and pathological functional states of the brain. But to compute above mentioned chaotic descriptors (quantifiers) it is necessary to reconstruct from raw EEG-data astrange attractor in multi-dimensional phase space. This takes relatively long time, needs high computing power and the results are difficult to comprehend for most of clinicians. On the other hand, analysis of Higuchi's fractal dimension does not require preliminary reconstruction of the phase space, is much quicker and more intuitive to apply.

Deterministic chaos could be regarded as a healthy flexibility of the human brain necessary for correct neuronal operations. Different functional states of the brain are probably governed by different degrees of complexity. Fractal dimension measures degree of complexity of EEG-signal, which reflects complexity of the underlying brain dynamics.

We have successfully applied running Higuchi's fractal dimension method for several other applications, like analysis of biosignals registered during sleep for sleep staging (both EEG-signals [31,32] and HRV-signals [33]), for vigilance (wakefulness) monitoring (cf. [22]), as well as for image analysis, e.g. for quality control of nanosensors' surfaces [34], and for SEM image analysis for roughness assessment of implant materials [35]. We are exploring and developing other nonlinear methods, in particular symbolic analysis methods (cf. [21,22]). Nonlinear dynamics methods may find application also in psychophysiology [36].

Our world is not governed by simple linear laws most of us were taught at school. If one man digs a ditch in one hour it does not mean that 60 men will dig the same ditch in one minute – this is obvious for everybody, even for those who do not know contemporary physics and mathematics. What in physics is called *nonlinearity *it is just stating the fact that elements forming a system do not act independently one from another – in the example above the system consists of one ditch and 60 men whose work is restricted by the size of the ditch. In biology and medicine two plus two often does not make four [37] – it is well known that the effect of a drug is not just simply proportional to its dose and interactions of different drugs are also extremely important. Chaos is revolutionary in that the overall approach requires us to adopt a different paradigm which, at times, may move us away from linear methods of data analysis.

## Appendix

### Higuchi's fractal dimension algorithm

Higuchi's algorithm (cf. [16]) calculates fractal dimension of a time series directly in the time domain. It is based on a measure of length, *L(k)*, of the curve that represents the considered time series while using a segment of *k *samples as a unit. If *L(k) *scales like

*L(k) *~ *k*^-*Df*^

the curve is said to show *fractal dimension D_*f *_*  because a simple curve has dimension equal *1 *and a plane has dimension equal *2 *value of *D*_*f  *_is always between *1 *(for a simple curve) and *2 *(for a curve which nearly fills out the whole plane). *D*_*f  *_measures *complexity *of the curve and so of the time series this curve represents on a graph.

From a given time series: *X(1), X(2),...,X(N) *the algorithm constructs *k *new time series:

*X*^*k*^_*m*_*: X(m), X(m + k), X(m + 2k),...,X(m + int((N-m)/k) · k) *for *m *= *1,2,...,k*

where *m *– initial time, *k *– interval time, *int(r) *– integer part of a real number *r*.

For example, for *k *= *4 *and *N *= *1000 *the algorithm produces 4 time series:

*X*^4^_1_*: X(1), X(5), X(9),...,X(997)*

*X*^4^_2_*: X(2), X(6), X(10),...,X(998)*

*X*^4^_3_*: X(3), X(7), X(11),...,X(999)*

*X*^4^_4_*: X(4), X(8), X(12),...,X(1000)*

The 'length' *L*_*m*_*(k) *of each curve *X*^*k*^_*m *_is then calculated as:

Lm(k)=1k⋅[(∑i=1int⁡(N−mk)|X(m+i⋅k)−X(m+(i−1)⋅k)|)⋅N−1int⁡(N−mk)⋅k]
 MathType@MTEF@5@5@+=feaafiart1ev1aaatCvAUfKttLearuWrP9MDH5MBPbIqV92AaeXatLxBI9gBaebbnrfifHhDYfgasaacH8akY=wiFfYdH8Gipec8Eeeu0xXdbba9frFj0=OqFfea0dXdd9vqai=hGuQ8kuc9pgc9s8qqaq=dirpe0xb9q8qiLsFr0=vr0=vr0dc8meaabaqaciaacaGaaeqabaqabeGadaaakeaacqWGmbatdaWgaaWcbaGaemyBa0gabeaakmaabmaabaGaem4AaSgacaGLOaGaayzkaaGaeyypa0ZaaSaaaeaacqaIXaqmaeaacqWGRbWAaaGaeyyXIC9aamWaaeaadaqadaqaamaaqahabaWaaqWaaeaacqWGybawcqGGOaakcqWGTbqBcqGHRaWkcqWGPbqAcqGHflY1cqWGRbWAcqGGPaqkcqGHsislcqWGybawcqGGOaakcqWGTbqBcqGHRaWkcqGGOaakcqWGPbqAcqGHsislcqaIXaqmcqGGPaqkcqGHflY1cqWGRbWAcqGGPaqkaiaawEa7caGLiWoaaSqaaiabdMgaPjabg2da9iabigdaXaqaaiGbcMgaPjabc6gaUjabcsha0naabmaabaWaaSaaaeaacqWGobGtcqGHsislcqWGTbqBaeaacqWGRbWAaaaacaGLOaGaayzkaaaaniabggHiLdaakiaawIcacaGLPaaacqGHflY1daWcaaqaaiabd6eaojabgkHiTiabigdaXaqaaiGbcMgaPjabc6gaUjabcsha0naabmaabaWaaSaaaeaacqWGobGtcqGHsislcqWGTbqBaeaacqWGRbWAaaaacaGLOaGaayzkaaGaeyyXICTaem4AaSgaaaGaay5waiaaw2faaaaa@7B5A@

where *N *– total number of samples.

*L*_*m*_*(k) *is not 'length' in Euclidean sense, it represents the normalized sum of absolute values of difference in ordinates of pair of points distant *k *(with initial point *m*). The 'length' of curve for the time interval *k*, *L(k)*, is calculated as the mean of the *k *values *L*_*m*_*(k) *for *m *= *1, 2,...,k*:

L(k)=∑m=1kLm(k)k
 MathType@MTEF@5@5@+=feaafiart1ev1aaatCvAUfKttLearuWrP9MDH5MBPbIqV92AaeXatLxBI9gBaebbnrfifHhDYfgasaacH8akY=wiFfYdH8Gipec8Eeeu0xXdbba9frFj0=OqFfea0dXdd9vqai=hGuQ8kuc9pgc9s8qqaq=dirpe0xb9q8qiLsFr0=vr0=vr0dc8meaabaqaciaacaGaaeqabaqabeGadaaakeaacqWGmbatdaqadaqaaiabdUgaRbGaayjkaiaawMcaaiabg2da9maalaaabaWaaabCaeaacqWGmbatdaWgaaWcbaGaemyBa0gabeaakmaabmaabaGaem4AaSgacaGLOaGaayzkaaaaleaacqWGTbqBcqGH9aqpcqaIXaqmaeaacqWGRbWAa0GaeyyeIuoaaOqaaiabdUgaRbaaaaa@3FD0@

The value of fractal dimension, *D*_*f  *_is calculated by a least-squares linear best-fitting procedure as the angular coefficient of the linear regression of the log-log graph of (1)

*y *= *ax *+ b¯
 MathType@MTEF@5@5@+=feaafiart1ev1aaatCvAUfKttLearuWrP9MDH5MBPbIqV92AaeXatLxBI9gBaebbnrfifHhDYfgasaacH8akY=wiFfYdH8Gipec8Eeeu0xXdbba9frFj0=OqFfea0dXdd9vqai=hGuQ8kuc9pgc9s8qqaq=dirpe0xb9q8qiLsFr0=vr0=vr0dc8meaabaqaciaacaGaaeqabaqabeGadaaakeaadaqdaaqaaiabdkgaIbaaaaa@2E0A@

with *a *= *D*_*f*_, according to the following formulae:

Df=n∗∑(xk∗yk)−∑xk∑ykn∗∑(xk 2)−(∑xk)2
 MathType@MTEF@5@5@+=feaafiart1ev1aaatCvAUfKttLearuWrP9MDH5MBPbIqV92AaeXatLxBI9gBamXvP5wqSXMqHnxAJn0BKvguHDwzZbqegyvzYrwyUfgarqqtubsr4rNCHbGeaGqiA8vkIkVAFgIELiFeLkFeLk=iY=Hhbbf9v8qqaqFr0xc9pk0xbba9q8WqFfeaY=biLkVcLq=JHqVepeea0=as0db9vqpepesP0xe9Fve9Fve9GapdbaqaaeGacaGaaiaabeqaamqadiabaaGcbaGaemiraq0aaSbaaSqaaiabdAgaMbqabaGccqGH9aqpdaWcaaqaaiabd6gaUjabgEHiQmaaqaeabaWaaeWaaeaacqWG4baEdaWgaaWcbaGaem4AaSgabeaakiabgEHiQiabdMha5naaBaaaleaacqWGRbWAaeqaaaGccaGLOaGaayzkaaGaeyOeI0YaaabqaeaacqWG4baEdaWgaaWcbaGaem4AaSgabeaakmaaqaeabaGaemyEaK3aaSbaaSqaaiabdUgaRbqabaaabeqab0GaeyyeIuoaaSqabeqaniabggHiLdaaleqabeqdcqGHris5aaGcbaGaemOBa4Maey4fIOYaaabqaeaadaqadaqaaiabdIha4naaDaaaleaacqqGRbWAaeaacqqGGaaicqqGYaGmaaaakiaawIcacaGLPaaacqGHsisldaqadaqaamaaqaeabaGaemiEaG3aaSbaaSqaaiabdUgaRbqabaaabeqab0GaeyyeIuoaaOGaayjkaiaawMcaamaaCaaaleqabaGaeGOmaidaaaqabeqaniabggHiLdaaaaaa@6C14@

where *y*_*k *_= *ln L(k)*, xk=ln(1k)
 MathType@MTEF@5@5@+=feaafiart1ev1aaatCvAUfKttLearuWrP9MDH5MBPbIqV92AaeXatLxBI9gBaebbnrfifHhDYfgasaacH8akY=wiFfYdH8Gipec8Eeeu0xXdbba9frFj0=OqFfea0dXdd9vqai=hGuQ8kuc9pgc9s8qqaq=dirpe0xb9q8qiLsFr0=vr0=vr0dc8meaabaqaciaacaGaaeqabaqabeGadaaakeaacqWG4baEdaWgaaWcbaGaem4AaSgabeaakiabg2da9iabdYgaSjabd6gaUnaabmaabaWaaSaaaeaacqaIXaqmaeaacqWGRbWAaaaacaGLOaGaayzkaaaaaa@376E@, *k *= *k*_1_,....,*k*_*max*_, and *n *denotes the number of *k*-values for which the linear regression is calculated (*2 *≤ *n *≤ *k*_*max*_).

The standard deviation of *D*_*f  *_is calculated as:

SDf=n∗[∑yk2−Df∗∑xkyk−b¯∗∑yk](n−2)∗[n∗∑xk2−(∑xk)2]
 MathType@MTEF@5@5@+=feaafiart1ev1aaatCvAUfKttLearuWrP9MDH5MBPbIqV92AaeXatLxBI9gBaebbnrfifHhDYfgasaacH8akY=wiFfYdH8Gipec8Eeeu0xXdbba9frFj0=OqFfea0dXdd9vqai=hGuQ8kuc9pgc9s8qqaq=dirpe0xb9q8qiLsFr0=vr0=vr0dc8meaabaqaciaacaGaaeqabaqabeGadaaakeaacqWGtbWudaWgaaWcbaGaemiraqKaemOzaygabeaakiabg2da9maakaaabaWaaSaaaeaacqWGUbGBcqGH9aqpdaWadaqaamaaqaeabaGaemyEaK3aaSbaaSqaaiabdUgaRbqabaaabeqab0GaeyyeIuoakmaaCaaaleqabaGaeGOmaidaaOGaeyOeI0Iaemiraq0aaSbaaSqaaiabdAgaMbqabaGccqGHxiIkdaaeabqaaiabdIha4naaBaaaleaacqWGRbWAaeqaaOGaemyEaK3aaSbaaSqaaiabdUgaRbqabaGccqGHsisldaqdaaqaaiabdkgaIbaacqGHxiIkdaaeabqaaiabdMha5naaBaaaleaacqWGRbWAaeqaaaqabeqaniabggHiLdaaleqabeqdcqGHris5aaGccaGLBbGaayzxaaaabaWaaeWaaeaacqWGUbGBcqGHsislcqaIYaGmaiaawIcacaGLPaaacqGHxiIkdaWadaqaaiabd6gaUjabgEHiQmaaqaeabaGaemiEaG3aaSbaaSqaaiabdUgaRbqabaaabeqab0GaeyyeIuoakmaaCaaaleqabaGaeGOmaidaaOGaeyOeI0YaaeWaaeaadaaeabqaaiabdIha4naaBaaaleaacqWGRbWAaeqaaaqabeqaniabggHiLdaakiaawIcacaGLPaaadaahaaWcbeqaaiabikdaYaaaaOGaay5waiaaw2faaaaaaSqabaaaaa@6A3C@

where (cf. eq. (AP2) above)

b¯=1n(∑yk−Df∗∑xk)
 MathType@MTEF@5@5@+=feaafiart1ev1aaatCvAUfKttLearuWrP9MDH5MBPbIqV92AaeXatLxBI9gBaebbnrfifHhDYfgasaacH8akY=wiFfYdH8Gipec8Eeeu0xXdbba9frFj0=OqFfea0dXdd9vqai=hGuQ8kuc9pgc9s8qqaq=dirpe0xb9q8qiLsFr0=vr0=vr0dc8meaabaqaciaacaGaaeqabaqabeGadaaakeaacuWGIbGygaqeaiabg2da9maalaaabaGaeGymaedabaGaemOBa4gaamaabmaabaWaaabqaeaacqWG5bqEdaWgaaWcbaGaem4AaSgabeaaaeqabeqdcqGHris5aOGaeyOeI0Iaemiraq0aaSbaaSqaaiabdAgaMbqabaGccqGHxiIkdaaeabqaaiabdIha4naaBaaaleaacqWGRbWAaeqaaaqabeqaniabggHiLdaakiaawIcacaGLPaaaaaa@41AD@

with standard deviation

Sb¯=1n∗SDF2∗∑xk2
 MathType@MTEF@5@5@+=feaafiart1ev1aaatCvAUfKttLearuWrP9MDH5MBPbIqV92AaeXatLxBI9gBamXvP5wqSXMqHnxAJn0BKvguHDwzZbqegyvzYrwyUfgarqqtubsr4rNCHbGeaGqiA8vkIkVAFgIELiFeLkFeLk=iY=Hhbbf9v8qqaqFr0xc9pk0xbba9q8WqFfeaY=biLkVcLq=JHqVepeea0=as0db9vqpepesP0xe9Fve9Fve9GapdbaqaaeGacaGaaiaabeqaamqadiabaaGcbaGaem4uam1aaSbaaSqaaiqbdkgaIzaaraaabeaakiabg2da9maakaaabaWaaSaaaeaacqaIXaqmaeaacqWGUbGBaaGaey4fIOIaem4uam1aa0baaSqaaiabdseaenaaBaaameaacqWGgbGraeqaaaWcbaGaeGOmaidaaOGaey4fIOYaaabqaeaacqWG4baEdaqhaaWcbaGaem4AaSgabaGaeGOmaidaaaqabeqaniabggHiLdaaleqaaaaa@4FCF@

Higuchi's fractal dimension has a scaling feature. Multiplication of all amplitudes *X*^*k*^_*m *_by a constant factor, *c*, causes multiplication of the 'length' *L*_*m*_*(k) *by the same factor. Such multiplication does not change *D*_*f *_(cf. eq. (AP2) above):

ln⁡(L(k))=Df⋅ln⁡(1k)+(b¯+ln⁡(c))
 MathType@MTEF@5@5@+=feaafiart1ev1aaatCvAUfKttLearuWrP9MDH5MBPbIqV92AaeXatLxBI9gBaebbnrfifHhDYfgasaacH8akY=wiFfYdH8Gipec8Eeeu0xXdbba9frFj0=OqFfea0dXdd9vqai=hGuQ8kuc9pgc9s8qqaq=dirpe0xb9q8qiLsFr0=vr0=vr0dc8meaabaqaciaacaGaaeqabaqabeGadaaakeaacyGGSbaBcqGGUbGBdaqadaqaaiabdYeamnaabmaabaGaem4AaSgacaGLOaGaayzkaaaacaGLOaGaayzkaaGaeyypa0Jaemiraq0aaSbaaSqaaiabdAgaMbqabaGccqGHflY1cyGGSbaBcqGGUbGBdaqadaqaamaalaaabaGaeGymaedabaGaem4AaSgaaaGaayjkaiaawMcaaiabgUcaRmaabmaabaGafmOyaiMbaebacqGHRaWkcyGGSbaBcqGGUbGBdaqadaqaaiabdogaJbGaayjkaiaawMcaaaGaayjkaiaawMcaaaaa@4BEE@

The only parameter of Higuchi's algorithm is *k*_*max*_. We have demonstrated that the value *k*_*max *_= *8 *works o.k. for sampling frequency 128 Hz and the value *k*_*max *_= *15 *for sampling frequency greater than 200 Hz; we have also demonstrated several positive features of the method, e.g. low sensitivity to noise [26].

Higuchi's fractal dimension is a quantifier evaluated directly in the time domain, without reconstruction of a strange attractor in a multi-dimensional phase space. Unlike in the case of standard chaotic quantifiers, e.g. correlation dimension or Lyapunov exponents, calculation of Higuchi's fractal dimension does not require reconstruction of the phase space, so it requires only short time intervals – a window containing 100 data point is enough to calculate one value of Higuchi's fractal dimension.
